# Targeting dopamine receptor D2 as a novel therapeutic strategy in endometrial cancer

**DOI:** 10.1186/s13046-021-01842-9

**Published:** 2021-02-08

**Authors:** Stuart R. Pierce, Ziwei Fang, Yajie Yin, Lindsay West, Majdouline Asher, Tianran Hao, Xin Zhang, Katherine Tucker, Allison Staley, Yali Fan, Wenchuan Sun, Dominic T. Moore, Chang Xu, Yi-Hsuan Tsai, Joel Parker, Varun Vijay Prabhu, Joshua E. Allen, Douglas Lee, Chunxiao Zhou, Victoria Bae-Jump

**Affiliations:** 1 Division of Gynecologic Oncology, Department of Obstetrics and Gynecology, University of North Carolina at Chapel Hill, Chapel Hill, NC 27599 USA; 2 Department of Gynecologic Oncology, Beijing Obstetrics and Gynecology Hospital, Capital Medical University, Beijing, People’s Republic of China; 3 Lineberger Comprehensive Cancer Center, University of North Carolina at Chapel Hill, Chapel Hill, NC USA; 4 Department of Genetics, University of North Carolina, Chapel Hill, NC USA; 5 Oncoceutics, Philadelphia, PA USA; 6 Omic Insight, LCC, Durham, NC 27713 USA

**Keywords:** ONC201, DRD2, Endometrial cancer, Invasion, Proliferation

## Abstract

**Background:**

ONC201 is a dopamine receptor D2 (DRD2) antagonist that inhibits tumor growth in preclinical models through ClpP activation to induce integrated stress response pathway and mitochondrial events related to inhibition of cell growth, which is being explored in clinical trials for solid tumors and hematological malignancies. In this study, we investigated the anti-tumorigenic effect of ONC201 in endometrial cancer cell lines and a genetically engineered mouse model of endometrial cancer.

**Methods:**

Cell proliferation was assessed by MTT and colony formation assays. Cell cycle and apoptosis were evaluated by Cellometer. Invasion capacity was tested using adhesion, transwell and wound healing assays. LKB1^fl/fl^p53^fl/fl^ mouse model of endometrial cancer were fed a control low fat diet versus a high fat diet to mimic diet-induced obesity. Following tumor onset, mice were treated with placebo or ONC201. Metabolomics and lipidomics were used to identify the obesity-dependent effects of ONC201 in the mouse endometrial tumors. DRD2 expression was analyzed by immunohistochemistry in human endometrioid and serous carcinoma specimens. DRD2 mRNA expression from the Cancer Genome Atlas (TCGA) database was compared between the four molecular subtypes of endometrial cancer.

**Results:**

Increasing DRD2 expression in endometrial cancer was significantly associated with grade, serous histology and stage, as well as worse progression free survival and overall survival. Higher expression of DRD2 mRNA was found for the Copy Number High (CNH) subtype when compared to the other subtypes. ONC201 inhibited cell proliferation, induced cell cycle G1 arrest, caused cellular stress and apoptosis and reduced invasion in endometrial cancer cells. Diet-induced obesity promoted endometrial tumor growth while ONC201 exhibited anti-tumorigenic efficacy in the obese and lean LKB1^fl/fl^/p53^fl/fl^ mice. Metabolomic analysis demonstrated that ONC201 reversed the obesity-driven upregulation of lipid biosynthesis and reduced protein biosynthesis in obese and lean mice.

**Conclusion:**

ONC201 has anti-tumorigenic effects in endometrial cancer cells and a transgenic mouse model of endometrial cancer, and DRD2 expression was documented in both human serous and endometrioid endometrial cancer. These studies support DRD2 antagonism via ONC201 as a promising therapeutic strategy for endometrial cancer that has already demonstrated pharmacodynamic activity and clinical benefit in both serous and endometrioid endometrial cancer patients.

**Supplementary Information:**

The online version contains supplementary material available at 10.1186/s13046-021-01842-9.

## Background

Endometrial cancer (EC) is the most common gynecologic malignancy, and alarmingly, the frequency and mortality from EC continues to rise, in part due to the obesity epidemic [1]. In 2020, 65,620 new cases will be diagnosed, and 12,590 women will succumb to this disease [2]. Patients with advanced/recurrent EC are unlikely to be cured by surgery, conventional chemotherapy (paclitaxel + carboplatin is the standard 1st line treatment), radiation or a combination of these. In patients with prior exposure to platinum chemotherapy, further chemotherapy, endocrine therapy, targeted therapy, and/or immunotherapy have low response rates between 10 and 38% with long-term disease-free survival being rare [3–7]. Thus, novel treatments are desperately needed.

Interest in personalized medicine and targeted therapies continues to rise. The dopamine receptor pathway has emerged as a novel oncologic target due to its documented role in cellular proliferation pathways [8, 9]. Historically, modulation of the dopaminergic pathway has primarily focused on neurologic diseases or anti-psychotic therapy. However, a growing body of literature reveals increased mRNA and protein expression of the G protein-coupled dopamine receptor D2 (DRD2) in many tumors including breast, colon, pancreas, prostate, glioblastoma, lymphoma, and endometrial cancer [10–14]. Knockdown of DRD2 or inhibition with pharmacologic antagonists in glioblastoma, pituitary tumors, pancreatic and breast cancer cells leads to inhibition of proliferation and migration as well as anti-tumorigenic effects in number of different xenograft mouse models [11, 13–16].

Imipridones are a new class of anti-cancer compounds that target G protein-coupled receptors. ONC201, the first-in-class clinical imipridone, selectively and competitively antagonizes DRD2, induces the integrated stress response through activation of ClpP and results in activation of the tumor necrosis factor-related apoptosis-inducing ligand (TRAIL) pathway [17–20]. In vitro studies in lymphoma, leukemia, glioblastoma, colorectal cancer have shown ONC201 increases apoptosis and inhibits AKT/mTOR signaling, leading to a corresponding decrease in tumor growth in mouse models [17, 19]. This is significant particularly since PI3K/AKT signaling is often deregulated in both Type I (endometrioid histology) and II (serous histology) ECs [21]. We recently found that initial in vitro testing of serous EC cell lines with ONC201 resulted in significant inhibition of cellular proliferation, induction of apoptosis, as well as anti-metastatic effects [22]. The results from several phase I and II clinical trials showed that ONC201 is clinically active and exceptionally well-tolerated with favorable pharmacokinetics, pharmacodynamics and clinical benefit in advanced cancers including lymphoma, glioblastoma and ECs [17, 23–26]. ONC201 has demonstrated durable objective responses in patients with H3 K27M mutant gliomas and FDA has granted Fast TRACK designation to this investigational agent [26, 27]. The objective of this study was to investigate the expression of DRD2 in human EC specimens as well as to evaluate the anti-proliferative and anti-tumorigenic effects of DRD2 antagonist ONC201 in EC cell lines and a genetically engineered mouse model of EC.

## Methods

### Cell culture and reagents

Four EC cell lines, Ishikawa, ECC-1, HEC1A and KLE, were used for all experiments. The ECC-1 cells were maintained in RPMI 1640 medium with 5% fetal bovine serum (FBS). The Ishikawa and KLE cells were maintained in DMEM/F12 with 10% FBS. HEC1A cells were cultured in McCoy’s 5A with 10% FBS. All medium was supplemented with 100 U/ml of penicillin and 100 μg/ml of streptomycin. The cells were cultured in humidified 5% CO2 at 37 °C. ONC201 was obtained from Oncoceutics, Inc.

### MTT assay

The four cell lines were plated and grown in 96-well plates at a concentration of 4000 to 6000 cells/well for 24 h. The cells were subsequently treated with varying doses of ONC201 for 72 h. MTT (5 mg/ml, Sigma) was added to the 96-well plates at 5 μl/well, followed by an additional hour of incubation. The MTT reaction was terminated through the addition of 100 μl of DMSO. The results were read by measuring absorption at 570 nm with a microplate reader (Tecan, Morrisville, NC). Quest Graph™ IC50 Calculator (AAT Bioquest) was used to calculate IC50 value. The effect of ONC201 was calculated as a percentage of control cell growth obtained from DMSO treated cells grown in the same 96-well plates.

### Colony formation assay

The ECC-1 and KLE cells were seeded (1000 cells/well in 6-cm dishes) in regular growth medium overnight, and medium was replaced with fresh complete regular growth medium containing the indicated concentrations of ONC201 for 36 h. Cells were cultured at 37 °C for 14 days, with medium changes every third or fourth day. Cells were stained with 0.5% crystal violet, and colonies were counted under microscope.

### Analysis of apoptosis and cell cycle by Cellometer imaging

The ECC-1 and KLE cells were treated with ONC201 for 24 to 36 h in six well plates. For apoptosis analysis, the cells were collected and resuspended in 100 μl binding buffer containing Annexin V-FITC and 0.5 μl of propidium iodide for 15 min. For cycle analysis, the cells were harvested and fixed in a 90% methanol solution for 1 h. The cells were resuspended in RNase A solution for 30 min, followed by incubation with propidium iodide (PI) staining solution for 10 min. All samples were immediately measured by Cellometer (Nexcelom, Lawrence, MA) to identify apoptotic cells and assess cell cycle progression [22, 28]. The results were analyzed by FCS4 express software (Molecular Devices, Sunnyvale, CA).

### Adhesion and invasion assay

For the adhesion assay, 2.5 × 10^3^ cells were added in laminin-1 coated 96-well plates with varying concentrations of ONC201 at 37 °C for 1.5 h. After this time period, the cells were fixed by 5% glutaraldehyde. Adhered cells were stained with crystal violet, and 10% acetic acid was used to solubilize the dye. The absorbance was measured at 570 nm using a microplate reader. For the invasion assay, the cells were starved for 12 h and then seeded in the upper chambers coated with BME. The lower chambers were filled with regular medium plus ONC201 for 6 h at 37 °C. After Calcein AM solution was added into lower chamber for 30 min, the lower chamber plate was measured using a plate reader for reading fluorescence at EX/EM 485/520 nm.

### Wound healing assay

The ECC-1 and KLE cells were plated at 3 × 10^5^ cells per well in a 6-well plate for 24 h and then replaced with media with 0.5% charcoal stripped FBS for 12 h. A uniform wound was created through the cell monolayer using a 200 μl pipette tip. Cells were treated with ONC201 immediately after scratching. Photographs were taken at 0, 24 and 48 h after scratching, and the area of the scratch was analyzed with ImageJ software (NCI, Bethesda, MD). Percent closure was measured compared to 0 h, and fold change was determined from percent closure of treated compared to untreated.

### Reactive oxygen species (ROS) assay

The cells were seeded onto a 96-well black culture plate overnight and then treated with ONC201 at the indicated doses for 24 h. 10 μl DCF-DA (10 mM) was added into the media for 30 min. The fluorescence intensity was measured at EX/EM 485/530 nm using a fluorescence micro-plate reader.

### Western immunoblotting

The ECC-1 and KLE cells treated with ONC201 for 24–36 h. Cell lysates were prepared in RIPA buffer. Protein concentration was measured by BCA assay. Equal amounts of protein were separated by 10–12% gel electrophoresis and transferred onto a PVDF membrane. The membrane was blocked with 5% nonfat dry milk and then incubated with different primary antibodies overnight at 4 °C. The primary antibodies from Cell Signaling (Beverly, MA) used in this study included as follows: MCL-1, BCL-2, Phos-S6, Pan-S6, Phos-AKT, Pan-AKT, PERK, Bip, Erol-1, IRE1-a, PD-I, CDK4, CyclinD1, E-cadherin, VEGF and Slug. DRD2 and DRD5 antibodies were from Santa Cruze Biotechnology Inc. (Dallas, TX).). β-Actin and α-Tubulin were from Sigma (St. Louis, MO). β-Actin and α-Tubulin were used at 1:5000 dilution, and the rest antibodies were diluted: 1:1500. The membrane was then washed and incubated with a secondary peroxidase-conjugated antibody for 1 h after washing. Antibody binding was detected using SuperSignal™ West Pico on the ChemiDoc™ Image System (Bio-Rad). After developing, the membrane was stripped and re-probed using antibodies against β-actin or α-tubulin to confirm equal loading. Intensity for each band was measured and normalized to β-actin or α-tubulin as an internal control.

### LKB1^fl/fl^ p53^fl/fl^ transgenic mouse model of EC

The LKB1^fl/fl^ p53^fl/fl^ genetically engineered mouse model of EC was used in this study as described previously in detail [28]. All mice were handled according to protocols approved by University of North Carolina at Chapel Hill (UNC-CH) Institutional Animal Care and Use Committee (IACUC). To mimic diet-induced obesity (DIO), half of the mice were subjected to a high fat diet (HFD), while the other half were subjected to a low fat diet (LFD) (Research Diets, New Brunswick, USA) at 3 weeks age. Intrauterine Ad-Cre injections of LKB1^fl/fl^ p53^fl/fl^ mice were performed on the left side of uterus at 6–8 weeks of age to induce EC. The LFD and HFD mice were further divided into the vehicle or ONC201 (130 mg/kg, weekly, Oral gavage, 4 weeks) treatment groups. Each group included 15 mice. The animals were weighed weekly throughout the study. All mice were euthanized after 4 weeks of ONC201 or vehicle treatment. At sacrifice, endometrial tumors were weighed and blood samples were taken. Half of the endometrial tumor was snap-frozen and stored at − 80 °C, and the other half was fixed in 10% neutral-buffered formalin and paraffin embedded.

### Immunohistochemistry of endometrial tumor specimens

Triplicate cores were made of 118 endometrial tumors at our institution from hysterectomy specimens, and tissue microarrays were created. Immunohistochemical analysis DRD2 (1:300, Santa Cruze Biotechnology Inc.) was performed on 4-μmol/L sections of formalin-fixed, paraffin-embedded tissues using standard methodologies in UNC-CH Translational Pathology Laboratory Core. Individual slides were scanned using the Aperio™ ScanScope (Aperio Technologies, Vista, CA), and digital images were analyzed using Aperio™ ImageScope. Non-parametric tests and Cox regression analysis were used to correlate DRD2 expression with clinical outcomes.

The mouse endometrial tumor tissues were formalin-fixed and paraffin-embedded at the Animal Histopathology Core Facility at UNC-CH. Slides (5 μm) were first incubated with protein block solution (Dako) for 1 h and then with the primary antibodies for Ki-67 (1:400), phosphorylated-S6 (1:300), VEGF (1:800) and BCL-2 (1: 1200) for 2 h at room temperature. The slides were then washed and incubated with appropriate secondary antibodies at room temperature for 1 h. Further processing was carried out using ABC-Staining Kits (Vector Labs, Burlingame, CA) and hematoxylin. Immunohistochemistry slides were scanned by Motic (Houston, TX) and scored by ImagePro software (Vista, CA).

### TCGA RNA-Seq analysis

Using the TCGA data portal, we collected DRD2 RNA-Seq expression for differential gene expression analysis among the 371 EC samples. Information regarding bio-specimen procurement, data processing, quality control and normalization has been described by the Cancer Genome Atlas Research Network and can be reviewed on the TCGA open-access data portal (http://cancergenome.nih.gov). The RNA-seq gene expression level 3 data utilized in this study were TCGA normalized gene read counts, pre-corrected such that the 75th percentile of each patient’s set of gene expression measurements were normalized to a value of 1000. Using a multiple linear regression model for DRD2, we tested the association between DRD2 expression and EC genomic subtype, POLE ultramutated (POLE), microsatellite instability hypermutated (MSI), copy-number low (CNL) and copy-number high (CNH) [29].

### Metabolomics measurements

Endometrial tumors were analyzed from the four groups (*N* = 5/group) by Metabolon (Durham, NC) according to their standard protocols [30, 31]. Briefly, unbiased global metabolomic profiling was achieved using methanol extracts of tumor tissues normalized to tissue weight. Analysis of extracts consisted of either ultrahigh performance liquid chromatography (Waters Corporation, Milford, MA) coupled with tandem mass spectrometry (UHPLC/MS/MS; Thermo-Finnigan, San Jose CA) in positive and negative ionization modes, or via gas chromatography/MS analysis (Thermo-Finnigan). Metabolites in tumor tissues were positively identified by matching chromatographic retention time, mass and MS/MS fragmentation patterns to a reference library of over 2500 purified, authenticated biochemicals. Identification of known chemical entities was based on comparison to metabolomic library entries of purified standards based on chromatographic properties and mass spectra. Data are presented as relative measures of “scaled intensity” and median scaling to 1. Missing values were imputed with the minimum.

### Statistical analysis

Descriptive statistics were used to summarize data, particularly medians and inter-quartile ranges and means and standard deviations. Both parametric (Student’s t, ANOVA, and linear regression) and nonparametric (Wilcoxon, Kruskal-Wallis) methods were used. The Kaplan-Meier method was used to estimate the time-to event functions of progression-free survival (PFS) and overall survival (OS). The log-rank test was used for comparisons of these functions. Both SAS (version 9.4, Cary, NC) and GraphPad Prism (version 6, La Jolla, CA) statistical software packages were used. All tests were two-sided with *p* < .05 considered significant.

## Results

### DRD2 expression was associated with prognosis in EC patients

**Fig. 1:**
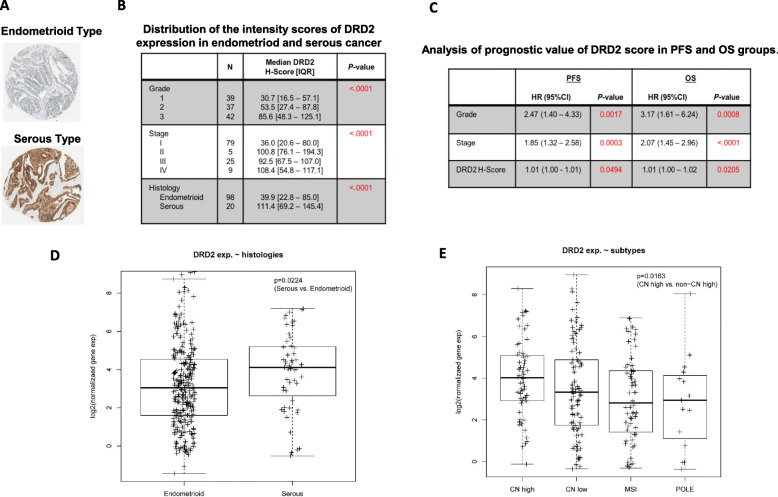
The expression of DRD2 in human ECs. The expression of DRD2 was detected by immunohistochemistry in 118 human tissues of serous and endometrioid ECs (**a**). Using median composite H-scores, DRD2 expression demonstrated statistically significant positive association with grade, stage, histologic types, PFS and OS in ECs (**b** and **c**). Identification of DRD2 mRNA expression from TCGA database in 371 human endometrial tumors based on histology (**d**) and molecular subtypes (**e**). High expression of DRD2 was found in serous-like copy-number high subtype when compared to the other subtypes. CN Low: Copy number low. CN high: Copy number high. MSI: Microsatellite Instability

A cohort of 118 human EC patients was identified that included 98 endometrioid and 20 serous histologies. Median patient age was 60.5 years with a median BMI of 31. Immunohistochemistry staining was performed for DRD2 in these tumors. Using median composite H-scores, increasing DRD2 protein expression was significantly associated with tumor grade (*p* < 0.0001), stage (p < 0.0001), and serous histology (p < 0.0001) (Fig. 1a and b). In addition, expression of DRD2 protein was significantly associated with progression free survival (PFS) (*p* = 0.049) and overall survival (OS) (*p* = 0.02, Fig. 1c). Using the TCGA database (*N* = 371 endometrial tumors), DRD2 mRNA expression was higher in serous type compared with endometrioid type EC (p = 0.02) (Fig. 1d). We further analyzed the expression of DRD2 distribution in molecular subtypes of EC based on molecular classification of endometrioid and serous carcinomas, and found high expression of DRD2 in serous-like copy-number high subtype when compared to the other subtypes (*p* = 0.005) as seen in Fig. 1e. These results confirm that DRD2 is expressed in both endometrioid and serous histology ECs; however, increased expression of DRD2 was associated with more aggressive histology’s (serous versus endometrioid) and molecular subtypes (copy-number high, CNH) as well as higher grade and stage.

### Effect of ONC201 on cell proliferation in EC cell lines

**Fig. 2:**
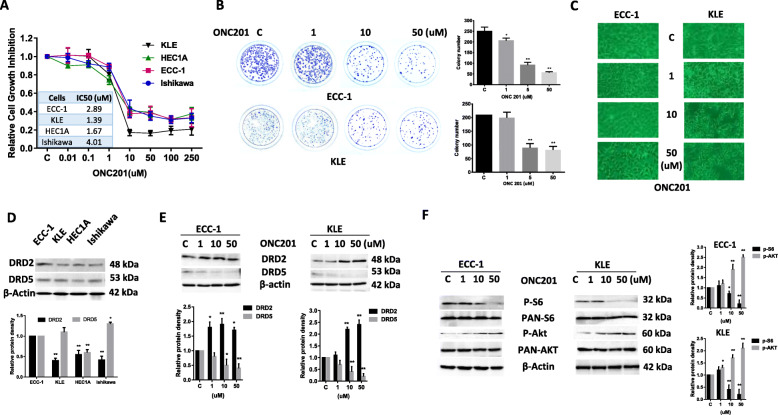
ONC201 induced dose-dependent growth inhibition in EC cell lines. Four endometrial EC cell lines, Ishikawa, ECC-1, HEC1A and KLE, were treated with various doses of ONC201 for 72 h. Cell proliferation was determined by MTT assay. Relative survival was determined by dividing the number of remaining ONC201 treated cells by the number of remaining viable DMSO (control). Representative dose-response curves and IC50 values are shown (**a**). The effect of ONC201 on the colony forming ability in ECC-1 and KLE cells was assessed through a colony-formation assay (**b**). The ECC-1 and KLE cells displayed distinct morphological alterations after 48 h treatment with ONC201 (**c**). Western blot analysis was performed to detect the expression of DRD2 in the four EC cell lines (**d**) and alternations of DRD2 and DRD5 after ONC201 treatment for 24 h in ECC-1 and KLE cell lines (**e**). The ECC-1 and KLE cells were cultured with various concentrations of ONC201 for 24 h followed by Western blot analyses to determine the expression of phosphorylation of AKT and S6. The results demonstrate that ONC201 inhibited the mTOR pathway in both cell lines (**f**). The levels of β-actin or α-tubulin served as protein loading controls. Data are representative of one of three independent experiments

Four EC cell lines, Ishikawa, HEC1A, ECC-1 and KLE, were treated with ONC201 at different concentrations for 72 h. Cell proliferation was assessed by MTT assay. The results showed that ONC201 demonstrated significant reduction in cell proliferation in a dose dependent manner in all cell lines. The IC50 dose was between 1.39 and 4.01 μM in all cell lines (Fig. 2a). Colony formation assays were performed to investigate the long-term effect of ONC201 on cell growth in the ECC-1 and KLE cell lines. As shown in Fig. 2b, the colony-forming ability of ECC-1 and KLE was reduced by 64 and 57% (*p* < 0.01), respectively, after exposure to 5 uM of ONC201 for 36 h and subsequent culture of cells for 14 days. The effects of ONC201 on cellular morphology in both cell lines are shown in Fig. 2c. Following ONC201 treatment for 48 h, both cell lines shrunk and displayed an elongated shape while control cells were round or oval shaped with large nuclei.

In order to determine if the level of DRD2 protein expression was related to the sensitivity to ONC201 in each cell line, we detected expression of DRD2 and DRD5 using western blotting in all four cell lines. The results revealed varying levels of DRD2 and DRD5 expression in the four cell lines. The expression levels of DRD2 and DRD5 were not associated with sensitivity to ONC201 in the cell lines. ECC-1 cells displayed the highest level of DRD2, but was not more sensitive to ONC201 than the other cell lines when compared with the IC50 for each cell line (Fig. 2d). Treatment of ECC-1 and KLE cells with ONC201 for 24 h significantly increased the expression of DRD2 and decreased the expression of DRD5 (Fig. 2e).

We next investigated whether the AKT/mTOR pathway was involved in the anti-proliferative effect of ONC201 in the ECC-1 and KLE cell lines. Western blotting showed that phosphorylation of S6 was repressed by ONC201 in a dose dependent manner after 24 h of treatment in both cell lines. ONC201 increased phosphorylation of AKT expression in the ECC-1 and KLE cells (Fig. 2f). Together, these results suggest that ONC201 inhibits cell proliferation in part via the AKT/mTOR/S6 pathway.

### Effect of ONC201 on apoptosis and cell cycle in EC cells

**Fig. 3:**
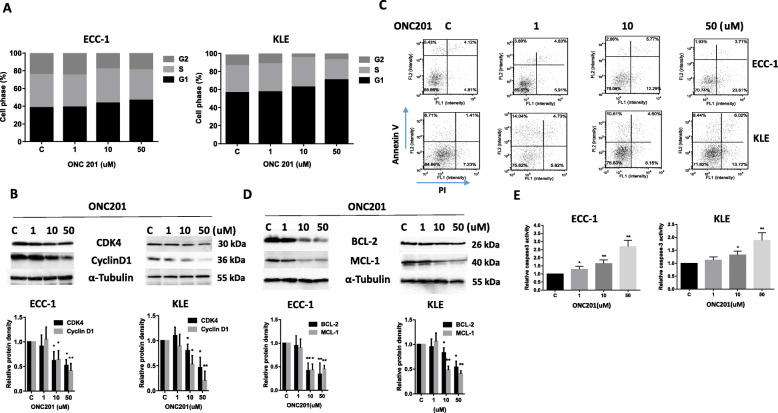
ONC201 induced cell cycle G1 arrest and apoptosis in EC cells. The ECC-1 and KLE cells were treated with various doses of ONC201 for 36 h. Cellometer analysis showed ONC201 induced cell cycle arrest in G1 phase in both cell lines (**a**). Cell lysates from the ECC-1 and KLE cell lines were subjected to Western blotting analysis for CDK4 and Cyclin D1 after treatment ONC201 for 24 h (**b**). Apoptosis was determined by Annexin V/ PI analyses after 24 h of treatment with ONC201 in ECC-1 and KLE cells. The profiles of Annexin V-FITC/PI staining are presented. The percentage of early apoptotic cells is located in the lower right quadrant (**c**). ECC-1 and KLE cells were cultured with different concentrations of ONC201 for 24 h, and then subjected to Western blot analysis on the levels of anti-apoptotic proteins. ONC201 reduced BCL-2 and MCL-1 expression in both cell lines (**d**). Data are representative of one of three repeats

To evaluate the underlying mechanism of growth inhibition by ONC201, the cell cycle profile was analyzed after treating the ECC-1 and KLE cell lines with varying doses (1-50uM) of ONC201 for 36 h. As illustrated in Fig. 3a, ONC201 induced G1 cell cycle arrest and reduced G2 phase in the ECC-1 and KLE cell in a dose-dependent manner. In the ECC-1 cells, G1 phase arrest increased from 36.26% in the control to 46.44% in cells treated with ONC201 at 50 μM (*p* < 0.05). In KLE cells, treatment with ONC201 increased G1 phase arrest from 56.36% in the control to 70.59% at a dose of 50 uM (*p* < 0.01). Western immunoblotting showed that ONC201 down-regulated the cell cycle related proteins, cyclin D and CDK4 (Fig. 3b). Thus, ONC201 treatment resulted in arresting cells in G1 phase in EC cells.

To further confirm whether the growth inhibition by ONC201 was related to apoptosis, we evaluated its apoptotic effect on ECC-1 and KLE cells by Annexin-V FITC stain analysis, which detects the phospholipid phosphatidylserine (PS) translocation from the inner (cytoplasmic) leaflet of the cell membrane to the external surface in very early apoptotic cells. As shown in Fig. 3c, after treatment of the cells with ONC201 at the indicated concentrations for 24 h, we found that ONC201 significantly increased early apoptotic cell populations in a dose dependent manner in both cells. Treatment with ONC201 (50 uM) induced 25.8% early apoptotic cells in ECC-1 and 13.7% in KLE cells. Western blotting results showed that ONC201 significantly reduced MCL-1 and BCL-2 expression in both cells after 24 h of treatment (Fig. 3d). To further explore the effect of ONC201 on the apoptotic pathways, an ELISA assay was used to detect the activity of cleaved caspase-3 in the ECC-1 and KLE cell lines treated with ONC201. We found that ONC201 increased cleaved caspase-3 activity in a dose-dependent manner after 24 h of treatment. Exposure of cells to 50 μM ONC201 resulted in 1.8 fold increase in cleaved caspase-3 activity in KLE and 2.7 fold increase in ECC-1 cells (Fig. 3e). These data suggest that ONC201 reduces proliferation through induction of apoptosis and G1 cell cycle arrest in EC cells.

### Effect of ONC201 on cellular stress in EC cells

**Fig. 4:**
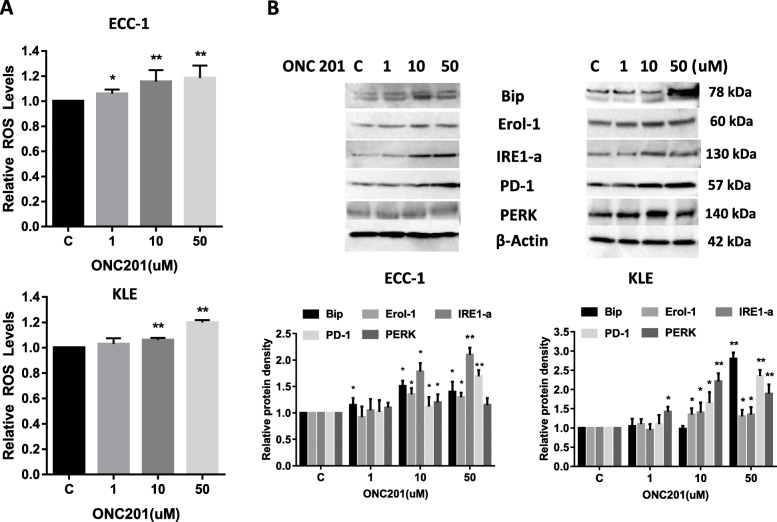
ONC201 induced cellular stress in EC cells. The ECC-1 and KLE cells were treated with ONC201 at indicated doses for 24 h and ROS production was assessed by DCFH-DA assay. ONC201 increased ROS production in a dose dependent manner in both cell lines (**a**). The cell lysates underwent Western blotting analysis for PERK, Bip, PD-1, Erol-1 and IRE1-a in both cells after 24 h treatment with ONC201. The results showed that ONC201 increased the expression of PERK, Bip, PD-1, Erol-1 and IRE1-a (**b**). The levels of α-tubulin served as protein loading controls. Data are representative of three or more independent experiments. *p < 0.05 and **p < 0.01

Reactive oxygen species (ROS) have been implicated in the cellular response to stress and are involved in mediation of apoptosis via mitochondrial DNA damage. To investigate the involvement of oxidative stress in the anti-proliferative effect of ONC201, intracellular ROS levels were examined by using the ROS fluorescence indicator DCFH-DA. As seen in Fig. 4a, treatment with ONC201 for 24 h significantly increased cellular ROS production in a dose-dependent manner in the ECC-1 and KLE cells. We next examined the changes of markers for endoplasmic reticulum (ER) stress after 24 h of ONC201 treatment in both cell lines. Western blotting showed ONC201 induced PERK, Bip, PD-1, Erol-1 and IRE1-a protein expression in a dose dependent manner, which is further evidence of ER stress induction by ONC201 (Fig. 4b). These results indicate that an increase in ROS might also be involved in the anti-tumorigenic effects of ONC201 in EC cells.

### Effect of ONC201 on adhesion and invasion in EC cells

**Fig. 5:**
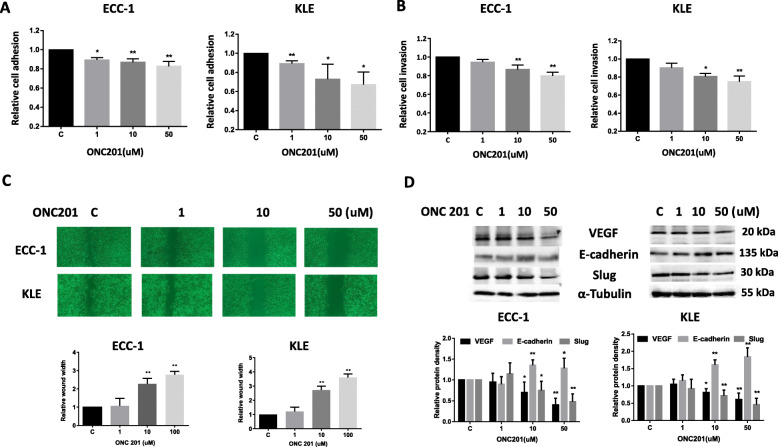
ONC201 reduced adhesion and invasion in EC cells. The ECC-1 and KLE cells were cultured with different doses of ONC201 for 90 min. Adhesion was determined by laminin-1 assay (**a**). Invasion was assessed by transwell assay in both cell lines after treatment with ONC201 for 6 h (**b**). Migration was measured by wound healing assay after treatment with ONC201 for 48 h (**c**). ONC201 significantly reduced the ability of adhesion, migration and invasion in both cell lines. Cell lysates from ECC-1 and KLE were subjected to Western blotting analysis for VEGF, Slug and E-cadherin after 24 h of treatment with ONC201 (**d**). ONC201 reduced the expression of VEGF and Slug and increased E-cadherin expression in both cell lines. The levels of α-tubulin served as protein loading controls. Results shown are representative of four independent experiment. *p < 0.05 and **p < 0.01

Adhesion and invasion of tumor cells are important steps leading to metastasis. In order to determine the effect of ONC201 on the invasive ability of EC cells, an in vitro laminin adhesion assay, transwell invasion system and wound healing assay were employed. Treatment of the ECC-1 and KLE cells with ONC201 for 90 min showed a significant reduction in adhesion in both cells (Fig. 5a). Exposure to ONC201 at concentrations ranging from 1 to 50 uM for 6 h significantly suppressed invasive ability of the cells compared to vehicle-treated control cells as determined by the transwell invasion assay (Fig. 5b). To examine the effect of ONC201 on motility in EC cells, we used a scratch wound healing assay to measure the extent of cell migration into the scratched area. ONC201 significantly slowed down cell migration into the “wounded” area in both cells after 72 h of treatment (Fig. 5c). To further analyze the effect of ONC201 on epithelial-mesenchymal transition (EMT) and vascular endothelial growth factor (VEGF) of EC cells, the levels of expression of E–cadherin, Slug and VEGF were analyzed by Western blotting. After 24 h of treatment, ONC201 increased expression of E-cadherin and decreased expression of slug and VEGF (Fig. 5d). Collectively, these results demonstrate that ONC201 inhibits the adhesion and invasion of EC cells.

### Anti-tumorigenic effect of ONC201 on LKB1^fl/fl^ p53^fl/fl^ mouse model of EC

**Fig. 6:**
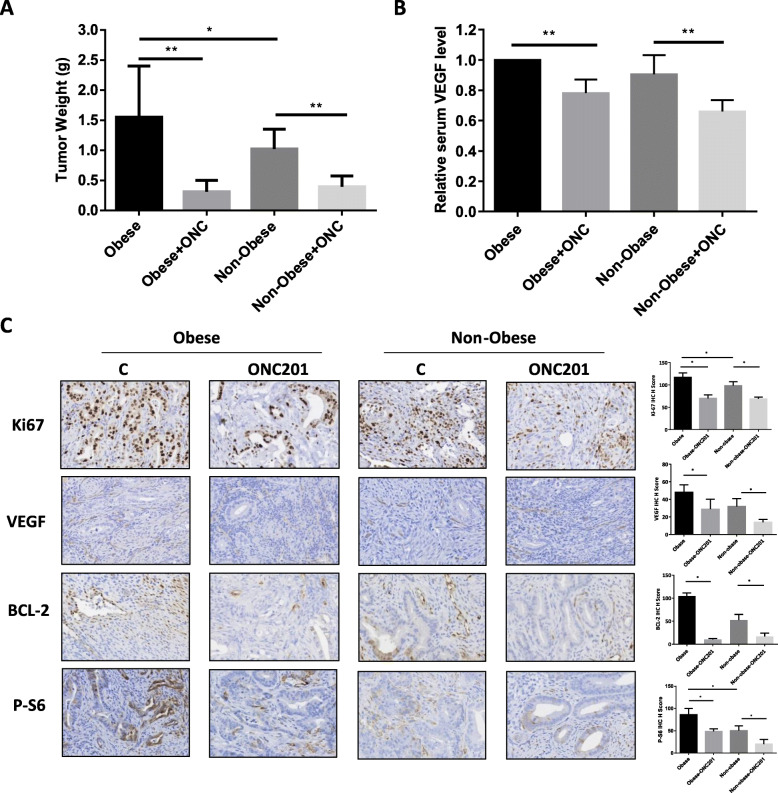
ONC201 inhibited tumor growth in the LKB1fl/fl/p53fl/fl mouse model of EC. The LKB1^fl/fl^ p53^fl/fl^ mouse genetically engineered mouse model of EC was used to evaluate the effect of ONC201 in vivo*.* The obese (HFD) or lean (LFD) mice were injected Ad Cre into left side of uterus at 6–8 weeks of age to induce ECs. After 8 weeks of injections, the mice were treated with ONC201 (130 mg/kg, oral, weekly) or vehicle for 4 weeks. The mean tumor weight was reduced in the ONC201 treatment groups in both obese and lean mice (**a**). Serum VEGF from each group was measured by ELISA assay (**b**). ONC201 decreased serum VEGF level in obese and lean mice treated with ONC201. The expression of KI67, VEGF, phosphorylated-S6 and BCL-2 was assessed using immunohistochemistry following ONC201 or placebo treatment in endometrial tumors under obese and lean conditions (**c**). ONC201 significantly reduced the expression of Ki67, VEGF, BCL-2 and phosphorylated-S6 in endometrial tumors under obese and lean conditions. *p < 0.05 and **p < 0.01

Given that ONC201 significantly inhibited EC cell proliferation and induced apoptosis in vitro, we sought to evaluate the effect of ONC201 on tumor growth in the LKB1^fl/fl^p53^fl/fl^ mouse model of EC. The mice were divided into four groups (15 mice/group) including LFD and HFD groups treated with either ONC201 or placebo. The mice were treated weekly by oral gavage with either ONC201 (130 mg/kg) or placebo for 4 weeks after tumor induction. This dose schedule mimics the administration of ONC201 in the clinic. The initial body weights of the obese mice at the starting treatment with ONC201 were 36.1 g, while that of the lean mice was only 26.2 g (*p* < 0.01, data not shown). Tumor weights were significantly increased in the control obese mice (HFD) compared to lean mice (LFD) at the completion of treatment, consistent with our previous work that found obesity promotes tumor growth in the LKB1^fl/fl^p53^fl/fl^ mouse model [28]. In the obese mice, tumor weight decreased by 79.1% (*p* < 0.01) with ONC201 treatment when compared with the obese control group. Among the lean mice, tumor weight decreased by 63.2% after treatment with ONC201 (p < 0.01) when compared with control-treated animals. Thus, ONC201 appeared to have a more pronounced impact on the tumor growth in obese mice (Fig. 6a). Although the average serum VEGF levels of lean mice is 10% lower compared with obese mice, no statistical significance was found between the two groups of mice. However, ONC201 significantly reduced the production of VEGF in serum following 4 weeks of treatment in both obese and lean mice compared to the controls (Fig. 6b).

Immunohistochemical analysis was performed on the endometrial tumors to assess effects of ONC201 on proliferation, angiogenesis, and downstream targets of the mTOR/S6 pathway. We found higher expression levels of Ki67 and phosphorylated-S6 in the tumor of obese mice than in that of lean mice, indicating that obesity promotes tumor proliferation and activates the mTOR pathway in endometrial tumors. The Ki-67 staining was significantly reduced by 51.8 and 34.5% in the obese and lean groups treated with ONC201 compared with the control treated mice, respectively (p < 0.01). Treatment with ONC201 significantly resulted in a decrease in the expression of phosphorylated-S6 and BCL-2 in both obese and lean mice, suggesting that ONC201 may result in downregulation of the mTOR pathway and induction of apoptosis in vivo. Importantly, we also found a significant decrease in the number of VEGF positive cells in the tumors of ONC201-treated obese and lean mice (Fig. 6c).

### Effect of ONC201 on metabolomic profiling in LKB1^fl/fl^ p53^fl/fl^ mouse model of EC

**Fig. 7:**
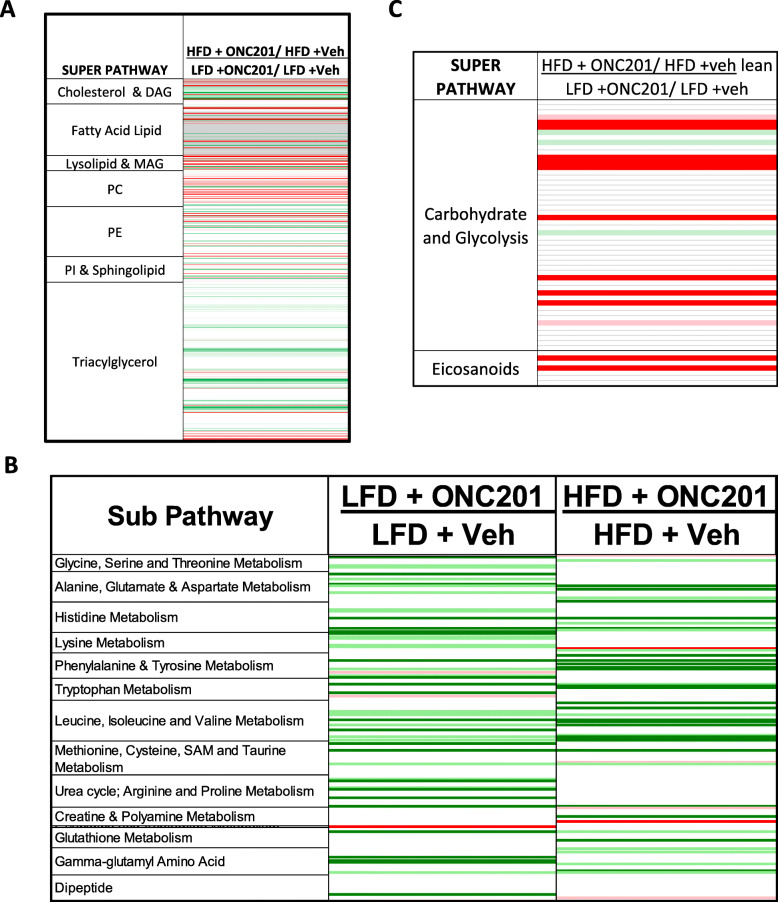
The effect of ONC201 on metabolites in the LKB1^fl/fl^/p53^fl/fl^ mouse model of EC. Metabolomic profiling were analyzed from the four groups (*N* = 5/group) by Metabolon. Endometrial tumors were found that lipid and protein biosynthesis were upregulated in the ECs of obese mice compared to lean mice. ONC201 increased lipid degradation and oxidation (**a**) and eicosanoid production in the ECs of obese mice but not lean mice (**b**). In contrast, ONC201 reduced glycolysis in the ECs of lean mice but not in the ECs of obese mice (**b**). ONC201 decreased protein biosynthesis in the ECs of both obese and lean mice (**c**). Red indicate metabolites with higher mean levels at a *p* ≤ 0.05 significance. Green cells indicate lower levels relative to control group at a *p* ≤ 0.05 significance. Data are presented as relative measures of “scaled intensity” and median scaling to 1

Metabolomic analyses of endometrial tumors from LKB1^fl/fl^p53^fl/fl^ mice revealed clear metabolic differences between obese and lean mice, also consistent with our previous work in this model [28]. To gain insight into the effect of ONC201 on metabolism in obese and lean LKB1^fl/fl^ p53^fl/fl^ mice, metabolic pathways of the significantly altered metabolites in endometrial tumors after treatment of ONC201 were determined using metabolomics and lipidomics. Metabolomic profiling revealed significant differences between obese and lean mice treated with ONC201 or placebo. ONC201 decreased availability of amino acids for protein biosynthesis in both obese and lean ECs. Lipid and protein biosynthesis were dramatically upregulated in ECs from obese control as compared to lean control mice. Treatment with ONC201 reversed the obesity-driven upregulation of lipid biosynthesis in the endometrial tumors, resulting in lipid degradation/oxidation as evidenced by decreased cholesterol esters and diacylglycerols/triacylglycerols and increased lysolipids in obese vs lean mice (Fig. 7a and b, Supplemental Fig. 1). ONC201 reduced glycolysis in the lean mice as evidenced in the significant decrease in dihydroxyacetone phosphate in lean tumors treated with ONC201 versus lean treated with placebo (*p* < 0.05). Glycolytic metabolites were not significantly altered in obese tumors receiving the same treatment (Fig. 7c). In addition, eicosanoids were increased in obese but not lean mice with treated ONC201, with over a 4-fold increase in 12-HHTRE and 6-keto prostaglandin F1, suggesting enhanced immune response in the setting of obesity (Supplemental Fig. 1 and 2).

## Discussion

Recent epidemiological studies found that patients with schizophrenia in which the dopaminergic pathway is activated have higher cancer risk, while the patients who received DRD2 antagonists treatment (e.g. haloperidol) have a reduced cancer risk in different solid tumors, including those of the rectum, colon, and prostate, suggesting that dopaminergic signaling pathways may be associated with the carcinogenesis of cancer [14, 32–34]. Multiple researchers have shown increased expression of dopamine receptors in malignancies [14, 17, 35]. Meredith *et. al.* identified frequent expression of DRD2 in neoplastic B cell populations, although treatment response with dopamine or dopamine-like compounds seemed independent of DRD2 expression [10]. Similarly, expression of DRD2 mRNA and protein was notably elevated in glioblastoma specimens relative to non-malignant cerebrum [11], and the pro-proliferative influence of DRD2 was mediated in part through the Ras/ERK signaling axis. Blockade of this pathway through DRD2 antagonism or gene silencing has in vitro and in vivo anti-proliferative effects in glioblastoma [11]. Recently, Jandaghi *et. al.* showed significant DRD2 mRNA and protein expression with corresponding activation of the DRD2 cell signaling pathway in human pancreatic tumors [14]. In addition, inhibition of DRD2 by siRNA reduced cell proliferation and migration, and slowed tumor growth in xenograft pancreatic cancer mouse models by inducing apoptosis and increasing cellular stress [14].

Targeting DRD2 with ONC201 has shown promising results in vitro and in vivo preclinical models in some types of cancers. Recent clinical trials showed that ONC201 has demonstrated well tolerated and durable objective responses in patients with H3 K27M mutant gliomas [26, 36]. Our recent study showed that ONC201 significantly inhibited cell proliferation, reduced ability of invasion and increased the sensitivity to paclitaxel in serous EC cell lines through inhibition of AKT/mTOR/S6 pathways [22]. In this study, we assessed the anti-proliferative, anti-metastatic and anti-tumorigenic effects of ONC201 in EC cell lines and mouse models. Treating EC cell lines with ONC201 inhibited cellular proliferation, induced ROS production and apoptosis, reduced cellular adhesion and invasion (Figs. 2, 3, 4 and 5). Unlike our recent results of ONC201 inhibiting AKT activity in serous EC cells, inhibition of mTOR/S6 pathway by ONC201 triggered a negative feedback loop, resulting in the activation of AKT signaling pathway (Fig. 2f) [22, 37]. Additionally, ONC201 significantly reduced tumor growth in a genetically engineered mouse model of endometrioid EC (LKB1^fl/fl^ p53^fl/fl^) under both obese and lean conditions, with a suggestion of greater effects seen in the setting of obesity (Fig. 6). Metabolomics and lipidomics results confirmed that ONC201 reversed the obesity-driven upregulation of lipid biosynthesis in the endometrial tumors, resulting in lipid degradation/oxidation as evidenced by decreased cholesterol esters and diacylglycerols/triacylglycerols and increased lysolipids in obese vs lean mice (Fig. 7). Lastly, this is the first study to demonstrate the correlation between the expression levels of DRD2 and the clinical and histopathological endpoints in human EC tumors [38]. Increased DRD2 expression in human EC tumors was significantly associated with PFS and OS, likely related to its correlation with grade, stage, and histology.

Given these data, intuitively one could expect higher DRD2 expression to lead to more sensitivity to ONC201 treatment; however, the high-DRD2-expressing cell line ECC-1 did not demonstrate increased sensitivity to ONC201 as compared to the other cell lines tested. We noted that treatment with ONC201 did increase the expression of DRD2 in this cell line (Fig. 2e), which potentially provided some proliferative advantage. Additionally, the complicated downstream effects of dopamine receptor pathways, the multiple potentially undescribed mechanisms of action of ONC201 and activation of pathways independent of DRD2 could explain this surprising result [39]. Prabhu *et. al.* recently found a DRD2 + DRD5- biomarker signature that significantly predicts the sensitivity to ONC201 in pre-clinical models and is associated with improved outcomes in patients treated with ONC201 in a Phase II clinical trial for recurrent glioblastoma [38]. Whether the level of DRD2 expression or DRD2 + DRD5- signature can be used as a predictive biomarker for response to ONC201 is an intriguing possibility and will be assessed in the ongoing clinical trials of ONC201 in both type 1 and 2 EC.

Using the TCGA database, we found expression of the DRD2 receptor was significantly increased in serous type or in the copy-number high subgroup. While the copy-number high subtype is comprised of primarily serous histology, it should be noted that 25% of high-grade endometrioid tumors fall into this aggressive copy-number high genomic subtype [29, 40]. Our immunohistochemistry results for DRD2 from our 118 EC patients further confirmed that higher DRD2 expression is associated with serous histology tumors (Fig. 1a-c). These findings suggest that the clinically aggressive nature of this subgroup may in part be related to activation of the dopaminergic pathway; and thus worthy of further exploration in future studies. We acknowledge that interpretation of this association may be limited by the retrospective nature of the data, small sample size and subjectivity of automated immunohistochemical scoring. However, this association makes antagonism of DRD2 a compelling treatment hypothesis for both type 1 and 2 endometrial tumors.

Targeting cancer metabolism seems to hold great promise to uncover novel targets for treatment, particularly in an obesity-driven cancer such as EC. Long-term antagonism of DRD2 with antipsychotic drugs significantly increases metabolic side effects including weight gain and disturbed lipid metabolism in patients with schizophrenia [41]. Recent studies have found that DRD2 signaling functionally alters glucose and lipid metabolisms in glioblastoma cells, and the effect of ONC201 on oxidative phosphorylation and glycolytic activity was dependent on genetic background of tumor cells [42, 43]. ONC201 treatment decreased oxidative phosphorylation through activation of ClpP to reduce basal oxygen consumption rate and enzymatic activity of respiratory chain complexes I, II, and IV in cancer cells [18, 20, 44]. We have previously found significant metabolic pathway differences between endometrial tumors from obese versus lean LKB1^fl/fl^ p53^fl/fl^ mice [28]. Thus, we postulated that the anti-tumorigenic effects of ONC201 may be accompanied by changes in glucose and lipid metabolism that are beneficial to cancer cell growth inhibition. Our metabolomics data showed that ONC201 significantly reduced glycolytic activity in endometrial tumors in the lean but not obese mice. Treatment of ONC201 resulted in a switch from obesity-driven upregulation of lipid biosynthesis to lipid degradation and oxidation; and thus, specific to only the obese mice. ONC201 treatment led to a reduction of protein biosynthesis, and this effect was shared between obese and lean mice (Fig. 7). These results clearly indicate that inhibition of lipid and protein biosynthesis could contribute to ONC201-mediated tumor suppression, and anti-tumorigenic effects of ONC201 may align with obesity status in vivo. Meanwhile, these results may also explain why ONC201 has an improved tumor suppressing effect in obese mice.

DRD2 has been shown to regulate many aspects of tumor behavior, including invasion and migration in cancer cells. Activation of DRD2 by the DRD2-specific agonist BIM53097 inhibits tumoral pituitary cells migration and invasion by activating the cofilin pathway in vitro [45, 46]. However, downregulation of DRD2 by trifluoperazine and haloperidol also results in decreased invasion and migration in prostate cancer cells, indicating the functional diversity of DRD2 and its downstream targets in different types of cancer [47]. TRAIL signaling, the integrated stress response, and the AKT/mTOR/S6 pathway are all known downstream targets of ONC201, and all have regulatory effects on cancer cell adhesion, migration and invasion [17, 22, 48–50]. Wagner *et. al.* found that ONC201 exerts a potent anti-metastasis effect via inhibition of cell migration and invasion in colorectal cancer cells and colon cancer mouse models [51, 52]. We also recently confirmed that ONC201 reduces adhesion and invasion in serous EC cell lines through induction of the epithelial-mesenchymal transition and inhibition of VEGF expression [22]. Similarly, in this current study, ONC201 exhibited an inhibitory effect on adhesion and invasion in EC cells and reduced VEGF production in both the serum and tumor tissues of the LKB1^fl/fl^ p53^fl/fl^ mouse model of EC (Figs. 5 and 6). Thus, future studies are planned to evaluate whether the anti-invasion effects of ONC201 are dependent on DRD2 signaling pathways versus other signaling effects.

Given that ONC201 has anti-tumor efficacy in preclinical models, the safety/tolerability and efficacy of ONC201 has been and is being tested in phase I and phase II clinical trials, respectively. Oral weekly ONC201 is well tolerated with very few adverse events and results in clinical activity in advanced solid tumors, including ECs [23, 53–55]. The unique features of ONC201 to occupy DRD2 and DRD3 at physiological concentrations strengthens the excellent safety profile of ONC201 in clinical trials [53]. Importantly, multiple ongoing phase I/II trials of ONC201 as a signal agent or in combination with other agents in solid tumors and hematologic malignancies has been initiated in the US. Needless to say, the results of clinical evaluation of ONC201 are highly anticipated in EC, where effective treatment options are limited.

## Conclusion

We found that ONC201 had efficacy in the inhibition of EC cell proliferation, tumor growth and metastatic potential in vitro and in vivo. Increased DRD2 expression was more common in human serous versus endometrioid ECs. These preclinical studies provide a fundamental rationale for investigating DRD2 antagonism with ONC201 in EC, with phase 2 clinical trials in type 1 and 2 endometrial cancer ongoing and which will hopefully mature in the upcoming year.

## Supplementary Information

**Additional file 1.**

**Additional file 2.**

## Data Availability

The datasets used in the current study are available from the corresponding author on reasonable request.

## Abbreviations

DRD2: Dopamine receptor D2
EC: Endometrial cancer
TCGA: The Cancer Genome Atlas
CNL: Copy Number Low
CNH: Copy Number High
EMT: Epithelial-mesenchymal transition
VEGF: Vascular endothelial growth factor
TRAIL: Tumor necrosis factor-related apoptosis-inducing ligand
FBS: Fetal bovine serum
HFD: High fat diet
LFD: Low fat diet
MSI: Microsatellite instability hypermutated
ROS: Reactive oxygen species
OS: Overall survival
PFS: Progression free survival
